# Primary jejunal melanoma as a cause of adult intussusception: a case report and review of literature

**DOI:** 10.11604/pamj.2019.33.214.18622

**Published:** 2019-07-16

**Authors:** Samuel Adegboyega Olatoke, Suleiman Olayide Agodirin, Adedire Timilehin Adenuga, Bashir Oladimeji Lawal, Kazeem Olatunde Ibrahim, Olaleke Oluwasegun Folaranmi

**Affiliations:** 1 Division of General Surgery, Department of Surgery, University of Ilorin Teaching Hospital, Ilorin, Kwara State, Nigeria; 2 Department of Pathology, University of Ilorin Teaching Hospital, Ilorin, Kwara State, Nigeria

**Keywords:** Jejunal melanoma, adult intussusception, case report, Nigeria

## Abstract

Primary melanoma of the small bowel is a rare clinical entity with a paucity of published reports in literature. Most cases of gastrointestinal melanomas are metastatic lesions arising from skin or ocular origins. This is a case report of a 63 year old female with adult intussusception with jejunal melanoma as the lead point. The index patient had a long history of abdominal pain associated with significant weight loss and presented with features of intestinal obstruction. The possibility of a regressed or unidentified extra-intestinal site cannot be absolutely excluded as the patient did not have a PET scan. Due to the vague nature of clinical symptoms and signs, the diagnosis of small bowel melanoma is difficult, especially in patients with no obvious cutaneous pathology. A high index of suspicion for melanoma as a malignant lead point for adult intussusception should always be entertained.

## Introduction

Melanoma is a malignant tumor originating from melanocytes which are usually located in the skin, the eye's choroid, the meninges, and the anal margin. They account for 1-3% of all intestinal tumours [1]. Primary melanoma of the small bowel is a rare clinical entity with a paucity of published reports in literature. Most cases of gastrointestinal melanomas are metastatic lesions arising from skin or ocular origins. The small bowel is frequently affected and is responsible for about 62% of malignant causes of small bowel intussusception [2]. The diagnosis of a primary gastrointestinal melanoma is clinically difficult because it is a diagnosis of exclusion where other common sources of metastasis must first be ruled out and most small bowel melanoma present with non-specific features such as abdominal pain, unexplained weight loss, gastrointestinal bleeding with features of anemia. Rare acute presentation may include intussusception and bowel perforation [3]. Clinically, adult intussusception remains an elusive diagnosis and accounts for only 1%-5% of intestinal obstructions in adults, with a pathologic lead point seen in up to 90% of cases [4]. There are several investigative modalities used in the diagnosis of uncomplicated small bowel melanoma which may include abdominal Computed Tomography (CT) and barium studies although they have low sensitivities. Video capsule endoscopy (VCE) is the gold standard as these lesions are usually beyond the reach of most conventional endoscopes. Surgery is the mainstay for intestinal melanomas and an oncologic resection should be done in resectable tumours [5]. Regardless of whether the melanoma is primary or secondary, intestinal melanomas are highly aggressive tumours, the prognosis is worse than cutaneous and other non-gastrointestinal melanoma. The survival rate at 5years is less than 10% [6]. This is a case report of a 63 year old female with adult intussusception with jejunal melanoma as the lead point. This is the first (to the best of our knowledge) reported case of adult intussusception caused by jejunal melanoma in Africa.

## Patient and observation

**Figure 1:**
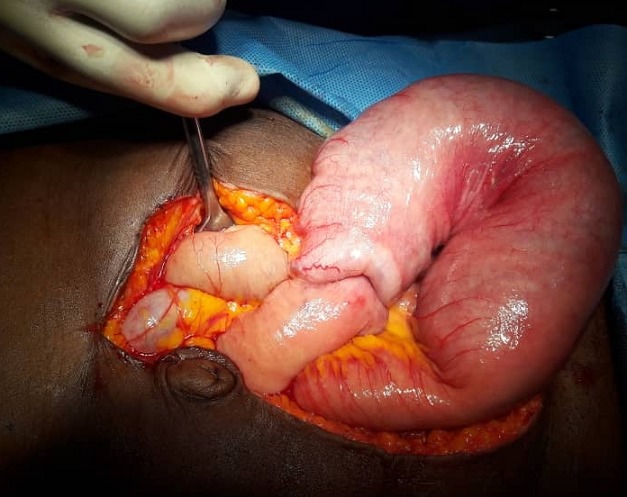
Showing the dilated proximal jejunum, intussusception and collapsed distal bowel

**Figure 2:**
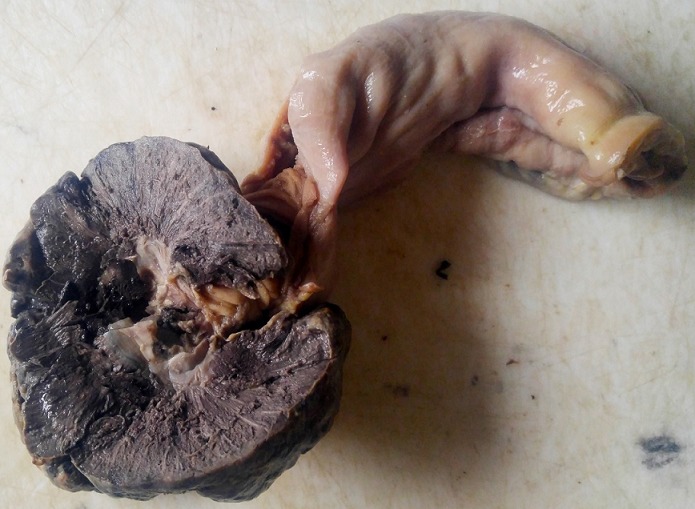
Gross picture of the resected jejunal segment showing a single black-grey mass arising from the jejunal wall

**Figure 3:**
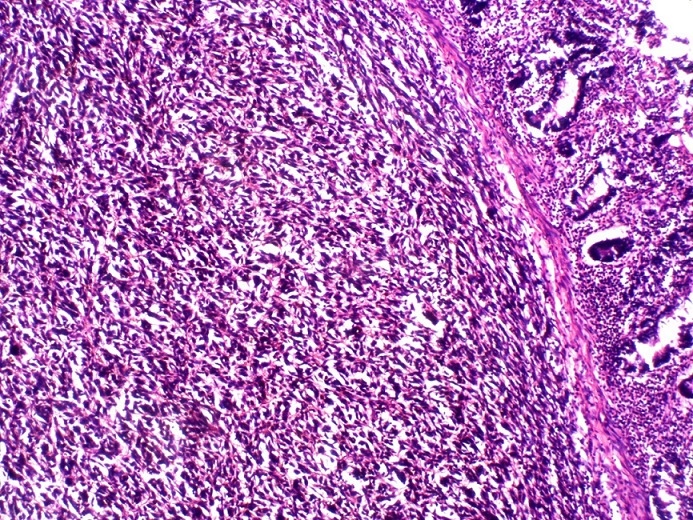
Histological section showing malignant spindle shaped cells in the submucosa. The overlying mucosa is intact. H&E x100

**Figure 4:**
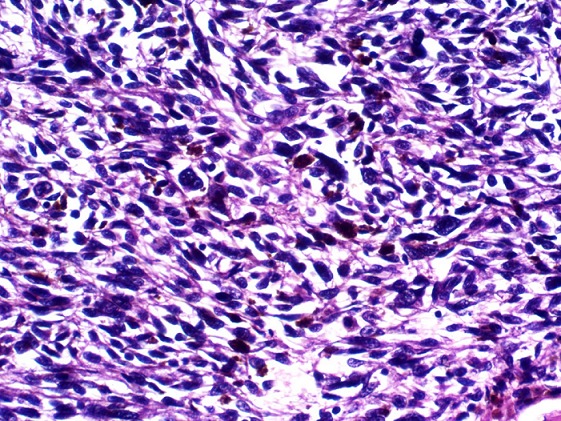
Histologic sections showing a higher power view of the malignant spindle melanocytic cells with marked nuclear atypia. Melanin pigment depositions are also evident, few are within melanophages. H&E x400

**Figure 5:**
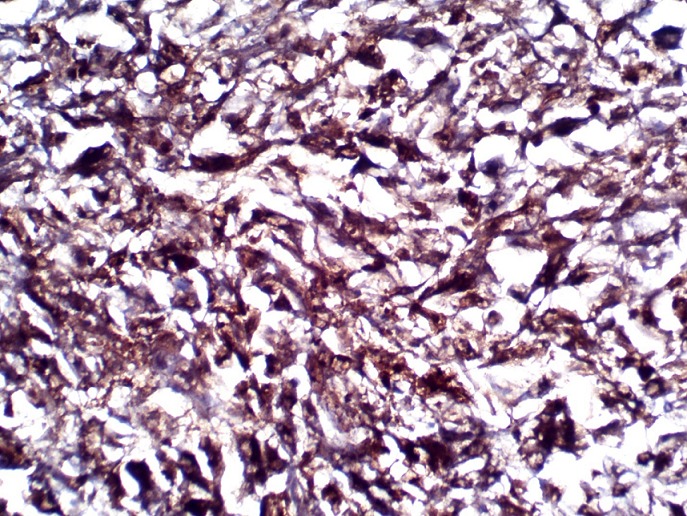
Immunohistochemical section showing cytoplasmic and nuclear positivity of the malignant cells for S100 protein. X400

A 63year old woman presented with recurrent central abdominal pain of four months duration, colicky, with postprandial bilious vomiting. There was associated weight loss and anorexia, generalized body weakness and dizziness. General physical examination revealed pallor without jaundice or lymphadenopathy. Abdominal, rectal examination and proctoscopy were unremarkable. Abdominal ultrasound done was suggestive of an intra abdominal mass with abdominal CT suggesting its location at the descending colon. Colonoscopy however did not show any masses. Complete blood count revealed anemia with a packed cell volume of 25%. She was transfused with two pints of blood preoperatively and worked up for surgery. Intra operative finding revealed a dilated proximal jejunum with collapsed distal aspect and a jejunojejunal intussusception about 80cm from the ligament of Trietz (Figure 1, Figure 2). This was excised en-bloc and sent for histology. Histology showed a lead point of melanoma. Extensive post operative clinical examination revealed no suspicion of melanoma. Pathology sections show small intestinal tissue with preserved mucosal lining, the subepithelium is characterized by infiltrating nodules of malignant spindle shaped cells extending from the submucosa to the muscular layers. The cells have large pleomorphic, hyperchromatic nuclei, prominent nucleoli and show brisk mitotic activity. There are florid areas showing melanin pigment deposition, with some showing phagocytosis by melanophages. The malignant cells show strong positivity for S-100 on immunohistochemistry (Figure 3, Figure 4, Figure 5). The resection margins are free.

## Discussion

Malignant melanomas are relatively common cancers making up around 2% of all tumors [7]. The vast majority of melanomas are cutaneous but non-cutaneous tumors occur albeit very rarely [7]. Malignant melanoma is also the commonest cancer to specifically metastasize to small bowel, comprising 50-70% of small bowel secondary cancers. The jejunum and ileum are most commonly involved. There is a report of melanomas in several body parts reported in Nigeria but this is the first report of a primary melanoma originating from the jejunum [8]. GI tract malignant melanoma is rare and may either represent metastasis from a primary cutaneous site or a true primary tumor arising from the GI mucosa. Certain experts believe that primary intestinal melanomas are derived from melanoblastic neural crest cells or from amine precursor uptake decarboxylase (APUD) cells that have undergone neoplastic transformation. Some proponents believe that primary small intestinal melanomas do not exist as a distinct clinical entity but are instead secondary deposits from a primary cutaneous melanoma which has either regressed or remained indolent and undiagnosed [9]. Infact some classify gastrointestinal melanomas without an obvious primary as Melanoma of Unknown Primary (MUP) failing to acknowledge that it could be a primary presentation of the aggressive disease. The use of Positron Emission Tomography (PET) scan in a bid to exhaustively look for possible primary sites of gastrointestinal melanoma has been recommended [2].

Differentiating between primary and secondary small bowel melanoma is challenging. Due to the vague nature of clinical symptoms and signs, the diagnosis of small bowel melanoma is difficult, especially in patients with no obvious cutaneous pathology. The symptoms may include gastrointestinal bleeding (melena, hematochezia, and occult blood), abdominal pain, vomiting, diarrhea, weight loss and asymptomatic anemia. Acute presentations with intussusception and perforation are rare; nevertheless, an awareness of these possibilities is important [3]. The index patient had a long history of abdominal pain associated with significant weight loss and presented with features of intestinal obstruction. Endoscopy and colonoscopy usually do not identify small intestine pathology and other ways of clinching the diagnosis must be considered, such as ultrasound, computed tomography, barium/technetium studies, positron emission tomography (PET) and capsule endoscopy. Most of the above listed investigative modalities are beyond the reach of the average patient in this environment. The index patient had a CT that wrongly suggested a descending colon mass, but colonoscopy was normal. Diagnosis was made at laparotomy.

Therapeutics in small bowel melanoma (SBM) is a field in need of development. Chemotherapy, immunotherapy and target therapy all have a role in medical treatment of SBM but they are almost invariably used palliatively. No systemic therapy is known to effectively treat intestinal melanomas and significantly improve survival [10]. Our patient did not receive any systemic adjuvant therapy. In setting of bowel obstruction, perforation or significant bleeding, emergency laparotomy for resection is mandatory. Resection of the affected intestine should be wide with suitable margins of normal bowel proximal and distal to the lesion, and should include resection of the associated affected mesentery and lymph nodes [1]. Manual reduction of the intussusception which is the gold standard treatment of pediatric intussusception is rarely a method of treatment in adults [4]. More often than not, the exact diagnosis may not be known and many workers advocate that resection of the intussuscepted segment be done without reduction in a bid to prevent spillage and also dissemination of tumour [4]. En-bloc resection reduces the possibility of recurrence and avoids repair/anastomosis on oedematous, ischemic bowel. A formal oncologic resection in patients above 60years with intussusception is recommended due to the possible high incidence of a malignant lead point which may approach 80% [4]. The index patient was above 60years and had en-bloc resection of the jejuno-jejunal intussusception done without reduction. The use of laparoscopy in the management of small bowel melanoma has been documented. It is largely dependent on proper patient selection and expertise of the surgeon. Laparoscopy rules out other differentials and provides a medium for possible intervention. Laparoscopy is still rudimentary in our setting and the index patient had a laparotomy done.

## Conclusion

In conclusion, primary SBM is a rare entity, which can be clinically difficult to diagnose in the setting of possible primaries at other places. The pathophysiology remains debatable. In our case, the possibility of a regressed or unidentified extra-intestinal site cannot be absolutely excluded as the patient did not have a PET scan. A high index of suspicion for melanoma as a malignant lead point for adult intussusception should always be entertained. A thorough clinical examination of the skin, eyes, anorectum, major lymph nodes and limbs should always be done once the histology is confirmed to find a primary. As with any malignancy, a timely and accurate diagnosis affords patients with more therapeutic options.

## Competing interests

The authors declare no competing interests.

## References

[1] Atmatzidis KS, Pavlidis TE, Papaziogas BT, Papaziogas TB (2002). Primary Malignant Melanoma of the Small Intestine: Report of a Case. Surg Today.

[2] Simons M, Ferreira J, Meunier R, Moss S (2016). Primary versus metastatic gastrointestinal melanoma: a rare case and review of current literature. Case Rep Gastrointest Med.

[3] Mucci T, Long W, Witkiewicz A, Mastrangelo MJ, Rosato EL, Berger AC (2007). Metastatic melanoma causing jejunal intussusception. J Gastrointest Surg.

[4] Kim JW, Lee BH, Park SG, Kim BC, Lee S, Lee S-J (2018). Factors predicting malignancy in adult intussusception: an experience in university-affiliated hospitals. Asian J Surg.

[5] Ait Idir B, Riany A, Jahid A, Chad B (2016). Primary melanoma of the small bowel revealed by gastrointestinal bleeding: a case report. J Med Case Rep.

[6] Kadivar TF, Vanek VW, Krishnan EU (1992). Primary malignant melanoma of the small bowel: a case study. Am Surg.

[7] Chang AE, Karnell LH, Menck HR (1998). The National Cancer Data Base report on cutaneous and noncutaneous melanoma: a summary of 84,836 cases from the past decade. The American College of Surgeons Commission on Cancer and the American Cancer Society. Cancer.

[8] Umobong E, Ojo B, Aghahowa M, Etim O, Ngbea J, Ugwu V (2014). Metastatic malignant melanoma presenting as small bowel obstruction: a report of a case. Am J Clin Med Res.

[9] Poggi SH, Madison JF, Hwu WJ, Bayar S, Salem RR (2000). Colonic melanoma, primary or regressed primary. J Clin Gastroenterol.

[10] Lens M, Bataille V, Krivokapic Z (2009). Melanoma of the small intestine. Lancet Oncol.

